# Analysis of high-dimensional metabolomics data with complex temporal dynamics using RM-ASCA+

**DOI:** 10.1371/journal.pcbi.1011221

**Published:** 2023-06-23

**Authors:** Balázs Erdős, Johan A. Westerhuis, Michiel E. Adriaens, Shauna D. O’Donovan, Ren Xie, Cécile M. Singh-Povel, Age K. Smilde, Ilja C. W. Arts

**Affiliations:** 1 Maastricht Centre for Systems Biology (MaCSBio), Maastricht University, Maastricht, The Netherlands; 2 Biosystems Data Analysis Group, Swammerdam Institute for Life Sciences, University of Amsterdam, Amsterdam, The Netherlands; 3 Dept. of Biomedical Engineering, Eindhoven University of Technology, Eindhoven, The Netherlands; 4 Netherlands Cancer Institute, Amsterdam, The Netherlands; 5 FrieslandCampina, Amersfoort, The Netherlands; University of Michigan, UNITED STATES

## Abstract

The intricate dependency structure of biological “omics” data, particularly those originating from longitudinal intervention studies with frequently sampled repeated measurements renders the analysis of such data challenging. The high-dimensionality, inter-relatedness of multiple outcomes, and heterogeneity in the studied systems all add to the difficulty in deriving meaningful information. In addition, the subtle differences in dynamics often deemed meaningful in nutritional intervention studies can be particularly challenging to quantify. In this work we demonstrate the use of quantitative longitudinal models within the repeated-measures ANOVA simultaneous component analysis+ (RM-ASCA+) framework to capture the dynamics in frequently sampled longitudinal data with multivariate outcomes. We illustrate the use of linear mixed models with polynomial and spline basis expansion of the time variable within RM-ASCA+ in order to quantify non-linear dynamics in a simulation study as well as in a metabolomics data set. We show that the proposed approach presents a convenient and interpretable way to systematically quantify and summarize multivariate outcomes in longitudinal studies while accounting for proper within subject dependency structures.

## Author summary

With advances in high-throughput omics platforms coupled with a reduction in associated costs, we increasingly see intervention studies generating extensive time-series of measurements simultaneously capturing changes across many dimensions. However, in order to derive meaningful information from such data, we must take into account the high-dimensionality, the interrelatedness of outcomes, the experimental design, the temporal dependency, as well as the subject-to-subject variability. Analytical tools that are able to account for all of these properties, in particular approaches that incorporate the temporal dependencies and the corresponding between-subject variability, are needed to make efficient use of such data. Here, we introduce novel methodology to quantify the temporal dependency and its subject-to-subject variability in high-dimensional, frequently sampled time-series data from longitudinal intervention studies. Our approach provides a systematic way to quantify and summarize multivariate outcomes in longitudinal intervention studies while accounting for the study design, the temporal dependency, and its between-subject variability. We demonstrate the effectiveness of this approach in a simulation study as well as on a metabolomics dataset.

## Introduction

The study of biological systems has seen enormous progress in recent decades in part due to the technological advances in the high-throughput data generating processes. These data often contain a large number of highly correlated variables frequently exceeding the number of samples. Moreover, the data may originate in various multi-factorial experiments carried out in heterogeneous populations. In particular, longitudinal interventions with repeated measures of multiple variables over time are used to generate time-series data to elucidate the dynamics and mechanisms of a system [1, 2]. However, to derive information from such data and assess the intervention effects, analysis must take into account the experimental design, the high-dimensionality, the heterogeneity in the population, and the correlatedness of the data both in terms of variables as well as time [3].

In the field of nutrition, experiments studying metabolic perturbations are commonplace. The dynamics of the metabolome are increasingly recognised as a more sensitive marker of metabolic health compared to fasting measurement and are regularly employed as indicators of intervention effects as well as pre-clinical and clinical conditions [4–6]. Standardised meal challenges are used to generate frequently sampled time-series data of metabolite concentrations in order to capture the dynamic alterations in the post-meal state [7]. The shapes of the resulting plasma metabolite transients are often non-linear and vary considerably across the study population [8]. Furthermore, nutritional intervention studies typically suffer from small effect sizes and sample sizes in addition to the high variation in the responses due to the heterogeneity of the underlying population [9]. All of these properties render it difficult to extract comprehensive information from such data.

Commonly used methodology to analyse longitudinal responses after meal challenges in nutritional interventions include univariate analysis, such as computing the area under the curve (AUC) or quantifying the change in time via linear mixed models (LMM) [10]. Multivariate analysis using ordinary differential equations (ODE) based models is also frequently employed [11]. While properties of the univariate analyses are well understood, they neglect the inter-relatedness of the outcomes and often require conservative false discovery rate (FDR) correction to report the univariate results. Moreover, many of the frequently used univariate techniques (e.g. AUC) disregard the dynamics of the outcome. Conversely, ODEs have been successfully used to describe the inter-relatedness of species as well as the dynamics in biological systems [12–14]. However, mechanistic models are often case-specific, and require prior knowledge or extensive and costly experiments to build and validate, therefore remain impractical to extend to high-dimensional scenarios. In addition, techniques within the functional data analysis (FDA) framework, such as functional PCA, have also been successfully applied in an exploratory fashion to analyse high-dimensional longitudinal data [15, 16].

Analytical approaches currently employed to this type of high-dimensional longitudinal data from designed interventions include extensions of the analysis of variance-simultaneous component analysis (ASCA) framework, a collection of methods based on decomposing the data matrix into additive effects and then performing principal component analysis (PCA) [17, 18]. Recently, this framework was extended to include random effects in linear mixed model-PCA (LiMM-PCA) and repeated measures-ASCA+ (RM-ASCA+) [19, 20]. These approaches work by estimating models of each metabolite time-course (in RM-ASCA+) or the (PCA) reduced score time-courses (LiMM-PCA). However, both methodologies considered simple longitudinal models with time as a qualitative factor, failing to capture the temporal dependency between time points. While this may be appropriate for comparison of before-after intervention effects or simple dynamics with few repeated measurements in time, time-as-factor models are not suited for frequently sampled time-series where the quantification of the temporal shape is important. In addition, application of such methods usually stops at quantifying the heterogeneity at baseline and rarely considers the heterogeneity in the temporal dynamics.

In this work we extend the RM-ASCA+ framework by introducing longitudinal linear mixed models with quantitative time variables in order to quantify multivariate outcomes over time. In addition, we also broaden the scope of the framework by accounting for heterogeneity in the temporal dynamics. We demonstrate that non-linear temporal effects can be recovered from noisy frequently sampled multivariate longitudinal data originating from a heterogeneous population using a simulation study. Then, we illustrate our approach on frequently sampled metabolomics data from the MELC Study, a double-blind, randomized, cross-over trial looking at the postprandial energy metabolism and lipemic response [21].

## 1 Methods

In this section, we first introduce the use of continuous time linear mixed models within the RM-ASCA+ framework through an example. Then, we describe the steps of analysis via RM-ASCA+ with such models to analyse frequently sampled multivariate longitudinal data. Finally, we specify the setup of two applications demonstrating the use of RM-ASCA+ using continuous time LMMs: a simulation study and analysis of a metabolomics data set.

### Longitudinal linear mixed model with continuous time

Suppose that response variable *y* was measured at *K* (*k* = 1, …, *K*) time-points in *I* (*i* = 1, …, *I*) subjects in a cross-over design where each subject underwent *H* (*h* = 1, …, *H*) treatments. In addition, we assume that the data displays quadratic profiles in time. If we consider the case where the number of treatments *H* = 2, the number of measurements *K* = 11, and the measurements are taken uniformly at ***t*** = (0, 1, 2, 3, 4, 5, 6, 7, 8, 9, 10) for both treatments in all individuals then a linear mixed model of the data can be written in the form:

$$\begin{array}{c} y_{ihk}=\left(\beta_{0}+\gamma_{i0}\right)+\left(\beta_{1}+\gamma_{i1}\right)\;t_{k}+\left(\beta_{2}+\gamma_{i2}\right)\;t_{k}^{2}+\left(\beta_{3}+\gamma_{i3}\right)\;t_{k}\;g_{h}+\left(\beta_{4}+\gamma_{i4}\right)\;t_{k}^{2}\;g_{h}+\epsilon_{ihk} \end{array}$$ (1)

where *β*_0−4_ are the fixed effects coefficients, *γ*_*i*0−4_ are random effects, *t*_*k*_ represents the sample time of the *k*^th^ measurement, *g* ∈ {−1, 1} is the indicator variable for treatment using sum coding, *t*_*k*_*g*_*h*_ and $t_{k}^{2}g_{h}$ are factor interactions between time and treatment and *ϵ*_*ihk*_ is a residual term. For simplicity, the example here demonstrates the use of linear and quadratic polynomials (i.e. the sample time *t* and its square) to introduce the temporal dependency. However, more generally, the temporal dependency may be coded by other basis functions (see section Modelling curvilinear trends in time). Sum coding of a factor variable leads to the effects of the factor levels being expressed relative to the mean across all groups. For more detail about the choice of coding and their interpretation we refer to [20]. The model per subject in matrix form can be written as:

$$\begin{array}{c} y_{i}=X_{i}\beta +Z_{i}\gamma_{i}+\epsilon_{i},\;i=1,\ldots ,I, \end{array}$$ (2)

where ***y***_*i*_ is a vector of length *HK*, ***X***_*i*_ and ***Z***_*i*_ are *HK* × *p* and *HK* × *q* design matrices, ***β*** and ***γ***_*i*_ are the vectors of fixed effects coefficients and random effects of length *p* and *q*, respectively, and ***ϵ***_*i*_ is the vector of residuals with length *HK*. The fixed effects parameterize the mean of the response, while the random effects allow the response trajectories in time to covary between individuals. The random effects and the residual error are assumed to have certain distributions that are specified through covariance matrix structures [22]. We assume, that $\gamma_{i}∼N_{q}\left(0_{q},D\right)$ with unstructured covariance matrix ***D***, and $\epsilon_{i}∼N_{HK}\left(0_{HK},\Sigma_{i}\right)$ with $\Sigma_{i}=I\sigma_{\epsilon }^{2}$ where ***I*** is the identity matrix and $\sigma_{\epsilon }^{2}$ is the residual variance. Additionally, we assume that ***γ***_1_, …, ***γ***_*I*_ and ***ϵ***_1_, …, ***ϵ***_*I*_ are independent. Since in our example the continuous time variable ***t*** is the same in both treatments for all subjects and the number of fixed and random effects coefficients *p* = *q* = 5, the design matrices take the form:

$$\begin{array}{l} \;int\;\;t\;\;\;\;\;t^{2}\;\;\;\;\;t\;g\;\;\;\;\;t^{2}g\\ X_{i}=Z_{i}=\left(\begin{array}{ccccc} 1 & 0 & 0 & 0 & 0\\ 1 & 1 & 1 & -1 & -1\\ 1 & 2 & 4 & -2 & -4\\ 1 & 3 & 9 & -3 & -9\\ \vdots & \vdots & \vdots & \vdots & \vdots \end{array}\right) \end{array}$$

The overall model (i.e. the model of all ***y***_*i*_’s) is then given by vertically stacking ***y***_*i*_, ***X***_*i*_, ***γ***_*i*_, and ***ϵ***_*i*_ for all *i*:

$$\begin{array}{c} \begin{array}{c} y=X\beta +Z\gamma +\epsilon ,\; \end{array}[\begin{array}{c} \gamma \\ \epsilon \end{array}]∼N([\begin{array}{c} 0_{Iq}\\ 0_{HK} \end{array}],[\begin{array}{cc} G & 0_{Iq\times HK}\\ 0_{HK\times Iq} & R \end{array}]) \end{array}$$ (3)

where vector ***y*** is of length *IHK*, the design matrices ***X*** and ***Z*** are *IHK* × *p* and *IHK* × *Iq*, respectively, ***β*** and ***γ*** are the vectors of fixed effects coefficients and random effects of length *p* and *Iq*, respectively, and ***ϵ*** is the vector of residuals of length *IHK*, with ***G*** = *diag*(***D***_1_, …, ***D***_*I*_), ***R*** = *diag*(**Σ**_1_, …, **Σ**_*I*_), and ***Z*** = *diag*(***Z***_1_, …, ***Z***_*I*_) block-diagonal matrices. The linear mixed models were implemented in R, ver. 4.0.2 using lme4, ver. 1.1–27.1 [23, 24]. The variance and covariance parameters defining ***D*** and **Σ** were estimated via restricted maximum likelihood estimation (REML).

### RM-ASCA+

Assume that instead of the single response variable in Eq 3, we have measured *J* (*j* = 1, …, *J*) response variables. In RM-ASCA+ Eq 3 is then extended to the multivariate case:

$$\begin{array}{c} Y=XB+Z\Gamma +E \end{array}$$ (4)

where ***Y*** is the *IHK* × *J* response matrix with *J* response variables, ***B*** is a *p* × *J* fixed effects parameter matrix, **Γ** is an *Iq* × *J* matrix of random effects, and ***E*** is a *IHK* × *J* residual matrix. To estimate ***B*** and the variance-covariance parameters specifying **Γ**, a LMM based on the design matrices ***X*** and ***Z*** is applied to every column of ***Y*** separately, then the coefficients and random variables are collected in ***B*** and **Γ**, respectively. The response matrix ***Y*** can now be decomposed into effect matrices by multiplying the corresponding columns of ***X*** with the corresponding rows of ***B***, and the corresponding columns of ***Z*** with the corresponding rows of **Γ**. For example, the following operation is used to obtain the effect matrix of the fixed effects of the time factor:

$$\begin{array}{l} \;int\;\;\;\;t\;\;\;\;\;t^{2}\;\;\;t\;g\;\;t^{2}g\\ M_{T}^{f}=\left(\begin{array}{ccccc} 0 & 0 & 0 & 0 & 0\\ 0 & 1 & 1 & 0 & 0\\ 0 & 2 & 4 & 0 & 0\\ 0 & 3 & 9 & 0 & 0\\ \vdots & \vdots & \vdots & \vdots & \vdots \end{array}\right)\times \left(\begin{array}{ccc} \beta_{01} & \ldots & \beta_{0J}\\ \beta_{11} & \ldots & \beta_{1J}\\ \beta_{21} & \ldots & \beta_{2J}\\ \beta_{31} & \ldots & \beta_{3J}\\ \beta_{41} & \ldots & \beta_{4J} \end{array}\right) \end{array}$$

The *IHK* × *J* effect matrix $M_{T}^{f}$ contains the population level multivariate profiles in time. The effect matrices of the random effects are obtained analogously. In general, the response matrix can be decomposed into effect matrices pertaining to each term in the model, however, similarly to $M_{T}^{f}$, effect matrices can contain multiple effects. Here, we consider the effect matrices:

$$\begin{array}{c} Y=M_{0}^{f}+M_{T}^{f}+M_{TG}^{f}+M_{0}^{r}+M_{T}^{r}+M_{TG}^{r}+E \end{array}$$ (5)

where ***M***_0_, ***M***_*T*_, ***M***_*TG*_ are the effect matrices for baseline, time, and time-treatment interaction corresponding to the intercepts, the time terms, and the interaction terms, respectively as shown for $M_{T}^{f}$ above. The superscripts *f* and *r* denote effect matrices containing fixed or random effects, respectively, and ***E*** contains the residuals. After centering, multivariate analysis of the effect matrix is then carried out via principal component analysis (PCA). For example, PCA of $M_{T}^{f}$ is given by:

$$\begin{array}{c} M_{T}^{f}=T_{T}^{f}P_{T}^{{}^{\prime }f} \end{array}$$ (6)

where $M_{T}^{f}$ is a *IHK* × *J* effect matrix, $T_{T}^{f}$ is a *IHK* × *A*_*T*_ score matrix, and $P_{T}^{{}^{\prime }f}$ is a *J* × *A*_*T*_ loading matrix, *A*_*T*_ being the number of principal components used in describing $M_{T}^{f}$, and ′ denotes the matrix transpose. The score and loading matrices are then visualized to elucidate the variation in the effect matrix. The dimensions of the effect matrix determine the dimensions of the score and loading matrices, thereby effecting the mode of visualization. In general, the rows of the effect matrix contain the observations to be compared visually in the form of scores following PCA. For example, the analysis in Eq 6 yields the *IHK* × *A*_*T*_ score matrix $T_{T}^{f}$, which allows the visualization of the multivariate population differences between treatments in time for every PC separately. For brevity, we refer to this mode of visualization as a ‘score trajectory’ plot. However, the effect matrix can also be reshaped prior to PCA to facilitate a different mode of visualization by reshaping the effect matrix so that the dimensions for time *K* and treatment *H* end up in the columns. For example, consider the effect matrix of the random effects of time, $M_{T}^{r}$. Reshaping $M_{T}^{r}$ from *IHK* × *J* to *I* × *HKJ* and analysing via PCA leads to a score matrix that allows the visualization of the multivariate differences between individuals using a conventional score plot. This mode of visualization is particularly useful in the case of effect matrices containing random effects. We will refer to these plots as ‘individual’ score plots due to their use in highlighting the similarities and differences between individuals.

Principal component analysis is performed on the centered effect matrices via singular value decomposition (SVD) using the base R function svd. The score and loading matrices ***T*** and ***P***′ in Eq 6 are the standardized scores ($\sqrt{n-1}U$) and loadings ($VS/\sqrt{n-1}$), respectively, where *n* is the number of rows of the effect matrix, the columns of ***U*** and ***V***^*T*^ contain the left and right singular vectors, and ***S*** is the diagonal matrix of singular values in SVD.

### Model validation

Approximate 95% confidence intervals for the scores and loadings are constructed through non-parametric bootstrapping as described in [20]. Briefly, all the observations are used to estimate a reference RM-ASCA+ model and the score and loading estimates are collected. Then, a bootstrap sample is created and the model is re-estimated. Subsequently, orthogonal Procrustes analysis is used to rotate the loading matrix of the bootstrap sample towards the reference loading matrix, the score matrix is then rotated using the resulting rotation matrix from the Procrustes analysis. The procedure was repeated a 100 times and the 2.5^th^ and 97.5^th^ percentiles of the bootstrapped score and loading estimates are used as lower and upper bounds for the confidence intervals.

### Simulation study

Synthetic metabolite responses over *K* = 11 repeated measurements are generated for a population of *I* = 40 subjects undergoing a randomized cross-over trial with *H* = 2 treatments by specifying the fixed and random effects coefficients in the model:

$$\begin{array}{c} y_{ihk}=\left({\tilde{\beta }}_{0}+{\tilde{\gamma }}_{i0}\right)+\left({\tilde{\beta }}_{1}+{\tilde{\gamma }}_{i1}\right)\;t_{k}+\left({\tilde{\beta }}_{2}+{\tilde{\gamma }}_{i2}\right)\;t_{k}^{2}+\left({\tilde{\beta }}_{3}+{\tilde{\gamma }}_{i3}\right)\;t_{k}\;g_{h}+\left({\tilde{\beta }}_{4}+{\tilde{\gamma }}_{i4}\right)\;t_{k}^{2}\;g_{h}+{\tilde{\epsilon }}_{ihk} \end{array}$$ (7)

where the fixed effects coefficients ${\tilde{\beta }}_{0-4}$ are selected to encode particular orthogonal temporal profiles, and the random effects ${\tilde{\gamma }}_{0-4}$ are sampled from a multivariate random distribution using a covariance matrix of non-zero off-diagonal elements (i.e. correlated random effects). The indicator variable for treatment *g* ∈ {−1, 1}, and the continuous time vector ***t*** = (0, 1, …, 10). Finally, uncorrelated random noise ${\tilde{\epsilon }}_{ihk}$ is added to the model simulations to obtain the synthetic responses *y*_*ihk*_. In total, *J* = 25 metabolite responses are generated using Eq 7 in such a way, that the fixed effects coefficients ${\tilde{\beta }}_{0-4}$ vary across the *J* metabolites. The responses are then collected in the simulated multivariate response matrix ***Y*** with dimensions *IHK* × *J* for all *J* metabolites.

In order to assess whether the encoded multivariate effects can be recovered from the noisy data, we decompose the simulated response matrix without the error term into effect matrices according to:

$$\begin{array}{c} \tilde{Y}={\tilde{M}}_{0}^{f}+{\tilde{M}}_{T}^{f}+{\tilde{M}}_{TG}^{f}+{\tilde{M}}_{0}^{r}+{\tilde{M}}_{T}^{r}+{\tilde{M}}_{TG}^{r} \end{array}$$ (8)

here, the effect matrices $\tilde{M}$ contain the encoded (ground truth) multivariate effects and are used as a reference in the re-analysis of the simulated data including the noise. The noisy synthetic data are then analysed with RM-ASCA+ and the encoded and RM-ASCA+ estimated effect matrices are visualized to evaluate whether the encoded multivariate effects were successfully recovered.

### Effect of fat source on post-meal lipoprotein dynamics from the MELC Study

Metabolomics data from the MELC Study, a double-blind, randomized, cross-over trial was used in this work [21]. The study data includes postprandial measurements in twenty healthy male adults who consumed two test drinks on separate days with a washout period in-between. The drinks differed in fat source, but otherwise were iso-energetic and equivalent in nutrient composition. Plasma samples were taken *K* = 11 times, at the fasting state (*t* = 0), and 30, 60, 90, 120, 180, 210, 240, 270, 300, 330 minutes after meal ingestion. The subset of data used here contains concentrations of very low density and low density lipoprotein (VLDL and LDL) subclasses (*J* = 36, cholesterol esters [CE], free cholesterol [FC], triglycerides [TG] and phospholipids [PL] in particle sizes XS to XXL) that were analysed using a nuclear magnetic resonance based metabolomics platform [25].

Prior to the analysis, measurements below the detection limit were removed. Subsequently, the measurements were divided by the standard deviation of the baseline measurements (*t* = 0) per metabolite. This scaling ensures that metabolites with relatively large variances at baseline do not disproportionately influence the RM-ASCA+ results [26]. We assume that there is no variation at baseline between the treatments. The data was then analysed using RM-ASCA+ with continuous time LMMs as described above (Eqs 1–6). In this case, we used natural cubic splines with two degrees of freedom instead of polynomials in Eq 1. Natural cubic splines are piece-wise cubic polynomials with continuous first and second derivatives at the knots with linearity constraints imposed at the tails of the boundary knots [27]. Natural splines are generally better behaved and do not suffer from the non-locality of polynomials at the same degrees of freedom [28, 29].

### Modelling curvilinear trends in time

There is a wide range of transformations that may be used to move beyond linearity besides the simple polynomials and splines demonstrated in this work. The choice of basis function depends on properties of the data, required amount of flexibility as well as considerations for numerical stability. A more detailed look at basis functions is out of scope for the current study, however, for a comprehensive overview of basis functions and splines we refer the reader to [27, 29, 30]. The natural cubic splines were implemented with B-spline basis using the ns function from the splines library (ver. 3.6.2). For more details about spline functions in R we refer to [31].

## 2 Results

### Simulation study

**Fig 1:**
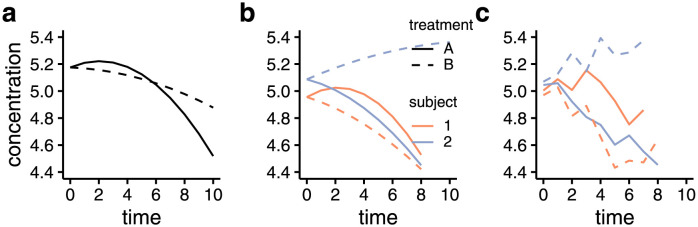
Example highlighting the process of generating an synthetic metabolite time-course. Panel *a* shows the population level metabolite time-courses determined by the fixed effects model parameters (${\tilde{\beta }}_{0-4}$ in Eq 7). In panel *b*, the population level curves are extended with subject-to-subject variability through the addition of random effects (${\tilde{\gamma }}_{0-4}$ in Eq 7). Finally, panel *c* contains the synthetic time-course with the added random noise. The response to treatment A and B are shown in continuous and dashed lines, respectively. Colours indicate the subjects to which the responses belong to. For parsimony, only two subjects are visualized.

Synthetic data containing 11 time-point time-courses of 25 metabolites after 2 treatments in a cross-over design were simulated in a population of 40 individuals. The resulting time-courses display varying rates of linear and quadratic changes over time and differences between the treatments across the metabolites in a heterogeneous population. The data generating process is highlighted through an example in Fig 1. First, the population level time-courses of a metabolite were specified through the fixed effects model parameters, then subject-to-subject variability was introduced through the addition of random effects. Importantly, heterogeneity in the dynamics was also added via the random effects corresponding to the time and time-treatment interaction effect. Finally, uncorrelated random noise representative of measurement noise was added to achieve the final simulated time courses. This process was repeated for all metabolites to create the synthetic data. All simulated time-courses are shown in S1 Fig.

**Fig 2:**
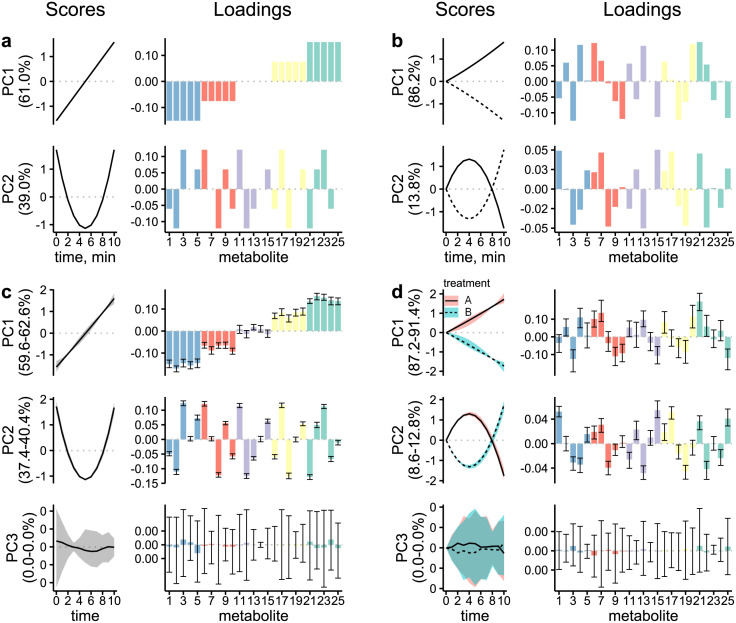
Simulation study design and results. The encoded (top row) and the RM-ASCA+ recovered (bottom row) effect matrices are summarized in score trajectory and loading plots. The columns correspond to the effect matrices for time (*a*, *c*) and time-treatment interaction (*b*, *d*), respectively. Scores contain the patterns in time (i.e. patterns over repeated measures) while the loadings indicate the association of score with the original metabolite time-course. The metabolites are coloured according to their PC1 loading magnitude for readability. Bootstrapped 95% confidence intervals are shown for the recovered effects as shaded area for the scores and error bars for the loadings.

The simulated synthetic data were collected into a multivariate response matrix and then decomposed into effect matrices containing the ground truth effects according to Eq 8. Subsequently, the multivariate effects in the simulated synthetic data were also estimated via RM-ASCA+. The resulting encoded (i.e. $\tilde{M}$) and corresponding estimated effect matrices (i.e. ***M***) were visualized via PCA to assess whether RM-ASCA+ with continuous time metabolite models could recover the encoded multivariate effects. The population level effect matrices are summarized visually as score trajectory and loading plots representing the encoded temporal patterns and their association with the original metabolite time-courses in Fig 2. The encoded time (${\tilde{M}}_{T}^{f}$) and time-treatment interaction (${\tilde{M}}_{TG}^{f}$) effect matrices are visualized in panels a and b, respectively, while the RM-ASCA+ estimated time ($M_{T}^{f}$) and time-treatment interaction ($M_{TG}^{f}$) effect matrices are shown on panels c and d. The encoded multivariate time and time-treatment interaction effects show a combination of slow increasing and parabolic trajectories in time (PC1 & PC2, panel a) as well as two distinct diverging patterns in the case of time-treatment interaction effects (PC1 & PC2, panel b). The most prominent temporal patterns explain 61% and 86.2% of the variance for the effect matrices of time and time-treatment interaction, respectively. The patterns across the loadings indicate the design according to which the fixed effects model parameters were specified in the data generating metabolite models.

In panels c and d of Fig 2, the RM-ASCA+ estimated score trajectory and loading plots of the population level time and time-treatment interaction effect matrices are shown. The estimated score trajectories and loadings show good agreement with the ground truth in panels a and b indicating that the encoded multivariate effects in the synthetic data could be recovered with RM-ASCA+ using the continuous metabolite models. Both the shapes of the prominent patterns in time (score trajectories) as well as their association with the metabolites (loadings) were conserved. In addition, PC3 shows that no artifact (effect outside of the encoded ground truth) was found using RM-ASCA+. The visualization in Fig 2 is representative of applying RM-ASCA+ to experimental data. Here, however, due to the simulated nature of the data, the ground truth scores and loadings are also known. Therefore, a direct comparison of encoded and estimated scores and loadings is shown in S2 and S3 Figs, respectively. In addition, the use of trailing PCs (such as PC3 in Fig 2) to indicate model validity only holds in the case when the ground truth is known.

**Fig 3:**
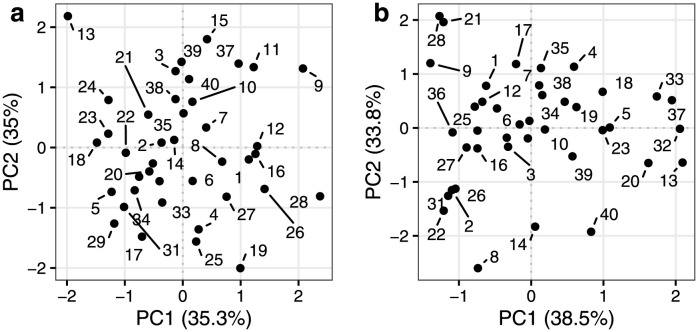
Score plot of the estimated effect matrices of (*a*) time, and (*b*) time-treatment interaction composed of the random effects model simulation. Each effect matrix was reshaped to *I* × *HKJ* prior to PCA. Points represent the simulated individuals.

The RM-ASCA+ estimated effect matrices of the random effects for time ($M_{T}^{r}$), and time-treatment interaction ($M_{TG}^{r}$) in Eq 5 were visualized in an individual score plot spanning PC1 and PC2 to facilitate the comparison of the simulated subjects’ responses in Fig 3. The score plots for the effect matrix of time (panel a) and time-treatment interaction (panel b) show that the subjects are randomly distributed around zero indicating agreement with the normal sampling distribution used for specifying the random effects. The origin is an approximation of the population average response profiles based on PC1 and PC2, therefore, the position of the subjects indicate how their responses differed compared to the population average. Additionally, the distance between the subjects is representative of how similarly they responded to each other. For example, given the score plot of time-treatment interaction (panel b), subjects 36 and 37 differ along their PC1 dimension indicating differential response in time to the two treatments. This was visually confirmed by looking at the synthetic responses in S4 Fig).

### Quantifying post-meal lipoprotein dynamics

**Fig 4:**
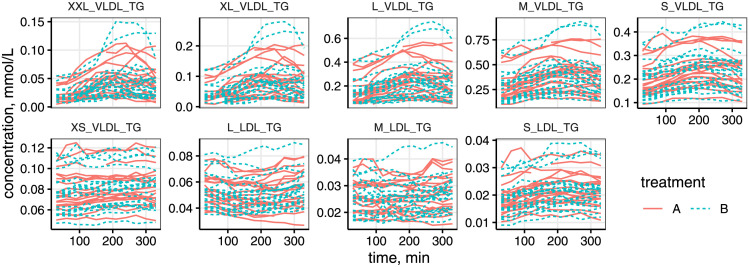
Triglycerides in very low and low density lipoproteins (VLDL, LDL) by particle size after the test drinks in the MELC Study. XXL: extra extra large, XL: extra large, L: large, M: medium, S: small, XS: extra small.

We applied RM-ASCA+ using continuous time LMMs on metabolomics data from the MELC Study. A subset of the post-meal lipoprotein responses used in the analysis including triglycerides (TG) in very low and low density lipoproteins (VLDL-LDL) after the test drinks is shown in Fig 4. The complete set of data used in the analysis is shown in S5 Fig.

After estimating the metabolite models we decomposed the multivariate response matrix into effect matrices according to the RM-ASCA+ framework as before in Eq 5. PCA analysis of the combined response matrix $M_{T}^{f}+M_{TG}^{f}$ summarizes the overall effect of the test drinks on the post-meal lipoprotein response in the population. Visualizing the results revealed a prominent slowly increasing pattern (PC1, 89.3–98.7% explained variance) that was primarily dominant in the VLDLs with decreasing prevalence going from XXL to S particle size. The XS VLDLs and the LDLs showed the inverse of the pattern denoted by the negative loadings. A faster responding component (PC2, 0.7–8.4% explained variance) was observed in L to S VLDLs and LDLs with the exception of TG in LDLs. The bootstrapped 95% confidence intervals suggest that there is no significant difference in the rate and shape of the multivariate responses between treatments A and B. PC3 contained no significant variation and was therefore discarded for interpretation.

The effect matrices $M_{T}^{f}$, and $M_{TG}^{f}$ can be examined in isolation to further elucidate the change in time, and the change in time due to the difference between the test drinks (S6 Fig). The change in time represents the average of the change induced by the two test drinks (panel a) due to the sum coding of the treatment effect. In addition, the additive nature of the effect matrices allows for insight into the relative variability between the sub-models. The confidence intervals in the score trajectory and loading plots indicate large variability within the population in the time-treatment interaction effects compared to the average change in time. In addition, no significant differences in the temporal profiles due to the test drink composition were found based on the time-treatment interaction results (panel b, S6 Fig).

**Fig 5:**
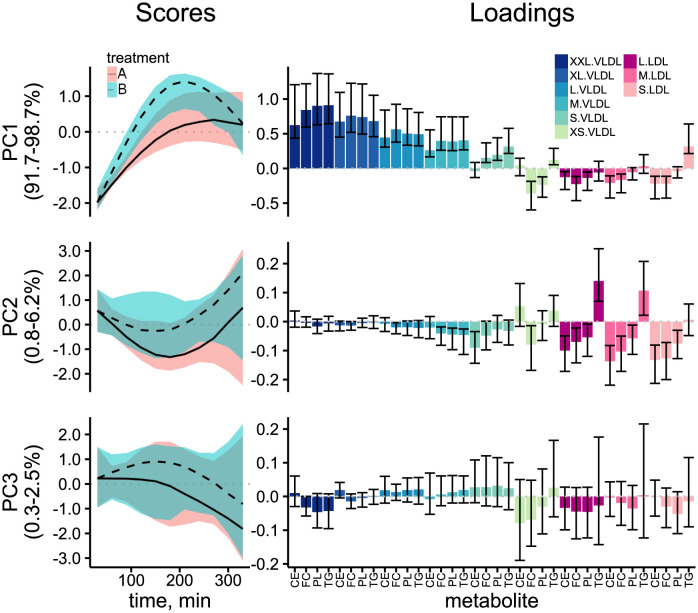
Score trajectory and loading plots of analysing the population level effect matrix for time+time-treatment interaction. Scores contain the predominent patterns over time, while the loadings show the association of the scores with the metabolite time-courses. Metabolites are shown in the axis label of the loadings with the colours indicating the various subclasses. FC: free cholesterol, CE: esterified cholesterol, PL: phospholipids, TG: tryglicerides. Bootstrapped 95% confidence intervals are shown as shaded area for the scores and error bars for the loadings.

**Fig 6:**
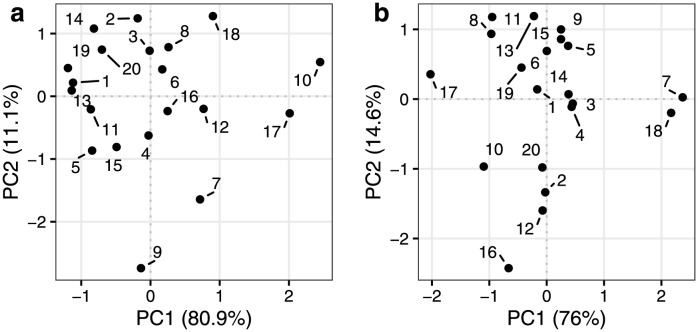
Individual score plot of PC1 and PC2 of the effect matrices of time (*a*) and time-treatment interaction (*b*) composed of the random effects model estimates. Points represent the individuals from the MELC Study.

The corresponding random effects model estimate-based effect matrices $M_{T}^{r}$ and $M_{TG}^{r}$ were consulted to elucidate the between-subject variability. First, PCA of the effect matrices in *IHK* × *J* orientation was carried out to show the individual-specific temporal profiles in a score trajectory and loading plot (S7 Fig). In addition, PCA was applied to the reshaped effect matrices (*I* × *HKJ*) leading to an individual score plot of the effect matrices to facilitate the comparison between individuals (Fig 6). The results summarise the heterogeneity in the post-meal dynamics of the population by showing how the individuals vary around their respective population level patterns (i.e. the results of the effect matrices composed of the fixed effects model estimates in Fig 5 and S6 Fig). In Fig 6, individuals close to the origin point responded similarly to the population level responses, while individuals further away showed diverging responses. The directions along the PC axes in Fig 6 correspond to the population level ones, e.g. individual 14 appearing to the left of the origin along the PC1 dimension relates to the participant’s lack of slow increasing response compared to those observed in the population trajectories in S6 Fig, panel b. In particular, the time-treatment interaction results showed that individuals within the population responded in opposite ways to the test drinks. Individual 7 generally had higher responses to treatment A compared to treatment B, while individual 17 responded to the contrary. These results were confirmed by consulting the metabolite responses in the data (S8 Fig).

## 3 Discussion

In this work, we extended the RM-ASCA+ framework towards frequently sampled multivariate time-series outcomes by introducing the use of LMMs with quantitative time as the underlying univariate temporal model. RM-ASCA+ is a highly flexible analytical framework for longitudinal multivariate data with multi-factorial experimental designs that yields easy-to-interpret and efficient representations of the study outcomes [20]. However, its applicability to studies where the shape of the longitudinal outcomes is of importance has been limited. We showed how RM-ASCA+ using LMMs with basis expansion of the numeric time variable may be used to capture the dynamics, including non-linearities, in the multivariate outcomes. Furthermore, we introduced and demonstrated the use of random effects in the models to allow the comparison of individual specific dynamics.

We illustrated the use and properties of RM-ASCA+ with quantitative time models by analysing synthetic multivariate time-courses representative of a heterogeneous population. The number of outcomes, sample size, and effect sizes were simulated to mirror the structure of dietary intervention studies in the field of nutrition. While the effect sizes and measurement noise were chosen to resemble real biological data, the encoded temporal effects (panels a and b, Fig 2) were selected to display notable orthogonal linear and quadratic profiles in order to make it easier to show the that the simulated effects were correctly estimated by the approach. In reality, such effects will not be orthogonal and therefore more difficult to interpret. Linear combination of the four encoded temporal effects of linear and quadratic changes in time as well as their interaction with the treatments can produce many heterogeneous shapes as shown in the simulated metabolites (S1 Fig). Additionally, the synthetic responses were simulated from LMMs including random effects pertaining to the temporal effects to introduce inter-individual variability in the dynamics of the responses. Using RM-ASCA+ with quantitative time models we were able to quantify the encoded population effects showing the potential in applying the approach on frequently sampled noisy data containing non-linear trajectories in a heterogeneous population.

Heterogeneity in the dynamics of the post-meal lipoprotein concentrations has been previously linked to functional differences in metabolism, while the postprandial lipoprotein profile was found to vary with factors such as gender and level of adiposity [32–34]. Frequently sampled postprandial Lipemic responses from the MELC Study have been previously analysed to quantify the post-meal dynamics after two test drinks in a population of healthy individuals [21]. However, the univariate analyses carried out in the original study did not account for the correlation across the metabolites even though they were measured in the same individual. A re-analysis of data from the MELC Study was carried out to demonstrate the novel RM-ASCA+ with quantitative time models which accounts for the multivariate nature of the data. The RM-ASCA+ derived representations provide a convenient view into the main modes of postprandial lipoprotein dynamics using the score trajectory and loading plots including typical patterns primarily determined by particle size as well as differential dynamics of triglycerides. In addition, the multivariate response profiles of individuals can be easily compared via the effect matrices of the random effects as shown in Fig 6.

A summary of the effect of the meal challenges on the lipoprotein responses in the MELC Study can be derived by visualizing the score and loading estimates of the combined time and time-treatment interaction effect matrices from RM-ASCA+ (Fig 4). The plots present a concise and convenient way to interpret as well as compare the various lipoprotein responses and treatments. In addition, the effect decomposition step allowed the quantification of the variability in the responses by effect source, highlighting the high heterogeneity in the responses to the test drinks by fat source. Furthermore, the underlying metabolite models were able to quantify between-subject variability in the dynamics via employing random effects of the time and time-treatment interaction effects. Thus, the approach allows insight into the inter-relatedness of the various lipoproteins as well as their changes in time, while accounting for subject-to-subject variability in the dynamics of the responses.

The approach outlined here allows the quantification of temporal dynamics while accounting for the dependency structures in data including within and between individual variability. Therefore, it presents an improvement over univariate methods frequently used to analyse post-meal dynamics such as AUC or LMM. Moreover, our approach is more easily generalizable and scalable to other systems than ODE-based models of postprandial dynamics, which may take a long time to build and validate.

The use of quantitative time models within RM-ASCA+ poses many benefits compared to the time-as-factor models demonstrated in [20]. Such approaches making use of polynomials or splines are frequently employed in univariate analysis to capture non-linearities in data. In particular, the use of explicit time models through various basis expansions of the numeric time variables allow the quantification of the dependency between the repeated measurements over time, making it possible to capture specific temporal shapes [35]. Additionally, as the number of repeated measurements in time grows, estimation of the time-as-factor models becomes less practical due to a large number of model parameters. Conversely, through the use of basis expansion methods such as polynomials and splines, quantitative time models present a flexible alternative with fewer parameters to estimate. Here, we demonstrated our approach using polynomial bases in a simulation study. However, it should be noted that the use of polynomial bases particularly ones with high order terms are generally not recommended due to ill-conditioning and their rigidity [28, 36]. These issues may be avoided by employing orthogonal polynomials or other basis expansions such as splines. Therefore, in the application to real data from the MELC Study, we used natural cubic splines. Quantification of the temporal dependency via continuous time models also supports the use of data with missing values. In addition, the sampling frequency, length of the sampled period, as well as the uniformity of the sampling scheme across subjects are also important in selecting the appropriate time model. A quantitative time model is flexible with regards to these properties and is generally favoured for data with irregular sampling strategies by subjects, as it requires no binning of the measurements or dropping them from the analysis.

A key step in RM-ASCA+ is selecting the model that will represent the change in the variables over time. This temporal model must be appropriately specified to quantify the change in the univariate models. Similarly to any univariate analysis via LMMs, this includes ensuring that the model appropriately represents the experimental setup, that the model parameters are identifiable, as well as examining model diagnostics [37–39]. In this work, the univariate model structure was shared across the metabolites i.e. all metabolites were modeled using the same LMM specification. Diagnostic plots of the residuals in the metabolite models fitted to the MELC Study are shown in S1 Appendix. While quantitative time models are quite flexible, care must be taken to avoid misspecification of the univariate models. For example, assume that most metabolites are appropriately described using a quadratic time variable, except for a particular metabolite that shows a delayed response. In such a case, the quadratic model would be misspecified; instead, a piece-wise model capturing the delay is necessary. The use of a model selection step within RM-ASCA+ to specify the model structure in each metabolite prior to multivariate analysis may be feasible, however was out of scope for the current work. Moreover, as heterogeneity in the longitudinal outcomes is a key feature of biological systems, we advocate the use of random effects of the model terms underlying the dynamics. Nevertheless, care should be taken when adding coefficients to estimate so that the model complexity is supported by the experimental design and data [39]. In principle, an arbitrary number of metabolites may be included in the analysis presented in this work, however, note that estimating the LMMs may be costly. In such cases, performing PCA on the metabolite responses first, as done in LiMM-PCA, may be a more scalable option [19, 20].

In conclusion, analysis of multi-outcome longitudinal data originating from multi-factorial experimental designs must take into account the within-subject dependency structures and account for the heterogeneity in the population. RM-ASCA+ is a novel analytical framework that takes into account the inter-relatedness of the multiple outcomes and population heterogeneity. Here, the framework is extended to quantify the dynamics in frequently sampled time-series data through the use of linear mixed models with numeric time predictors as the underlying univariate models of RM-ASCA+. Additionally, we showed how non-linearities in time, and heterogeneity in the dynamics -both frequently observed properties of biological systems- can be captured within RM-ASCA+ through the use of basis expansion, and random effects of the model terms describing the dynamics.

## Supporting information

S1 Fig Simulated metabolite responses. (TIF)

S2 Fig Encoded vs. RM-ASCA+ estimated scores of the fixed effects effect matrices of time and time-treatment interaction from the simulation study. Bars indicate resampling based 95% confidence intervals. Diagonal line represents perfect agreement.
(TIF)

S3 Fig Encoded vs. RM-ASCA+ estimated loadings of the fixed effects effect matrices of time and time-treatment interaction from the simulation study. Bars indicate resampling based 95% confidence intervals. Diagonal line represents perfect agreement.
(TIF)

S4 Fig Simulated metabolite responses with the responses of subjects 36 and 37 highlighted. (TIF)

S5 Fig Lipoprotein responses by particle size in the MELC Study [21]. XXL: extra extra large, XL: extra large, L: large, M: medium, S: small, XS: extra small.
(TIF)

S6 Fig Score trajectory and loading plots of analysing the effect matrices for time, and time-treatment interaction (panels a, and b, respectively). Scores contain the prominent patterns over time, while the loadings show the association of the scores with the metabolite time-courses. Metabolites are shown in the axis label of the loadings with the colours indicating the various subclasses. FC: free cholesterol, CE: esterified cholesterol, PL: phospholipids, TG: tryglicerides. Resampling based 95% confidence intervals are shown as shaded area for the scores and error bars for the loadings.
(TIF)

S7 Fig Score trajectory plot of the effect matrices time+time-treatment interaction, time, and time-treatment interaction composed of the random effects model estimates. (TIF)

S8 Fig Lipoprotein responses by particle size after the test drinks in 20 healthy young males in the MELC Study with highlighted individuals. XXL: extra extra large, XL: extra large, L: large, M: medium, S: small, XS: extra small.
(TIF)

S1 Appendix Diagnostic residual plots of the metabolite models fitted to the MELC study data. (7Z)
