# Supplementary figures and images for: Analysis of high-dimensional metabolomics data with complex temporal dynamics using RM-ASCA+
Source: PLoS Comput Biol. 2023 Jun 23;19(6):e1011221. doi: 10.1371/journal.pcbi.1011221 (PMC10325080; doi:10.1371/journal.pcbi.1011221)

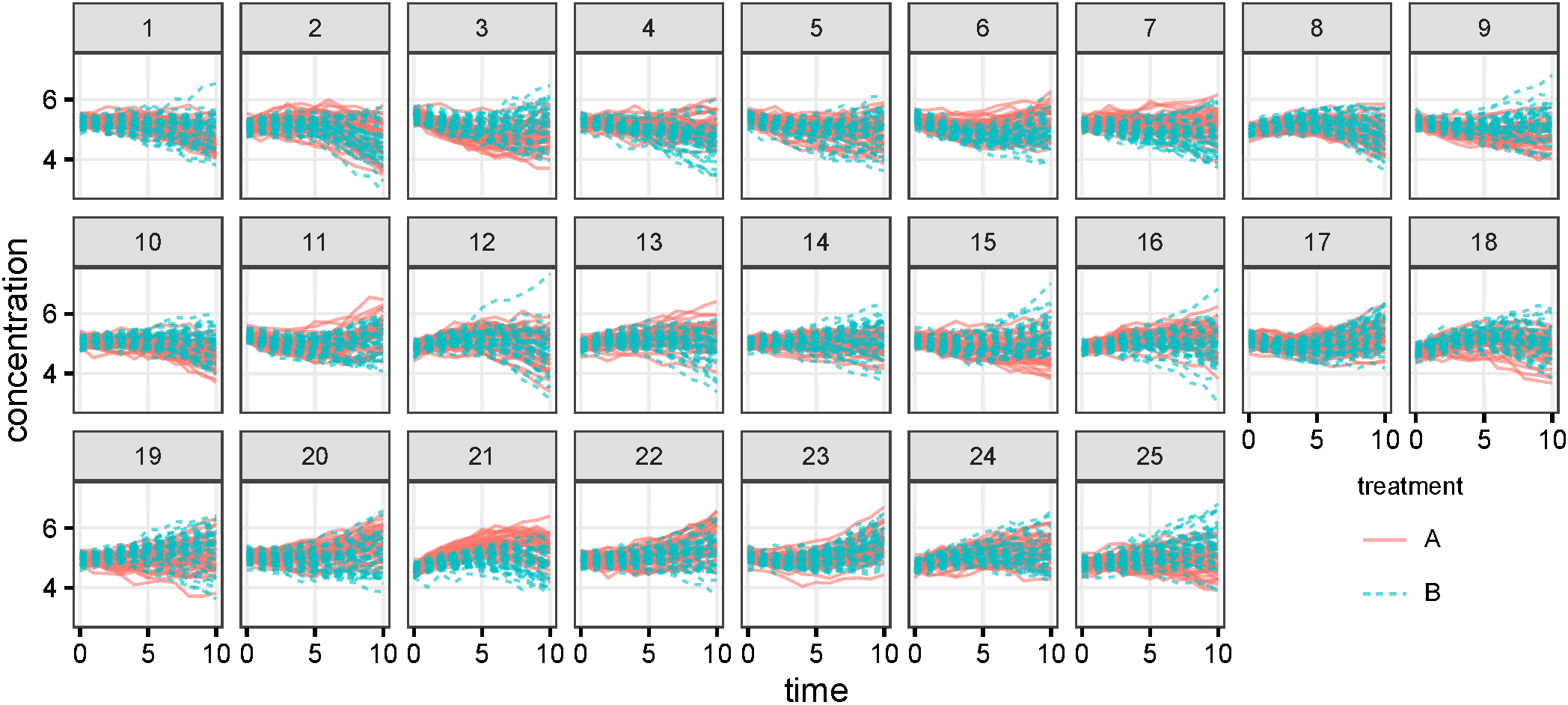

Supplement: S1 Fig — (TIF) [file pcbi.1011221.s001.tif]

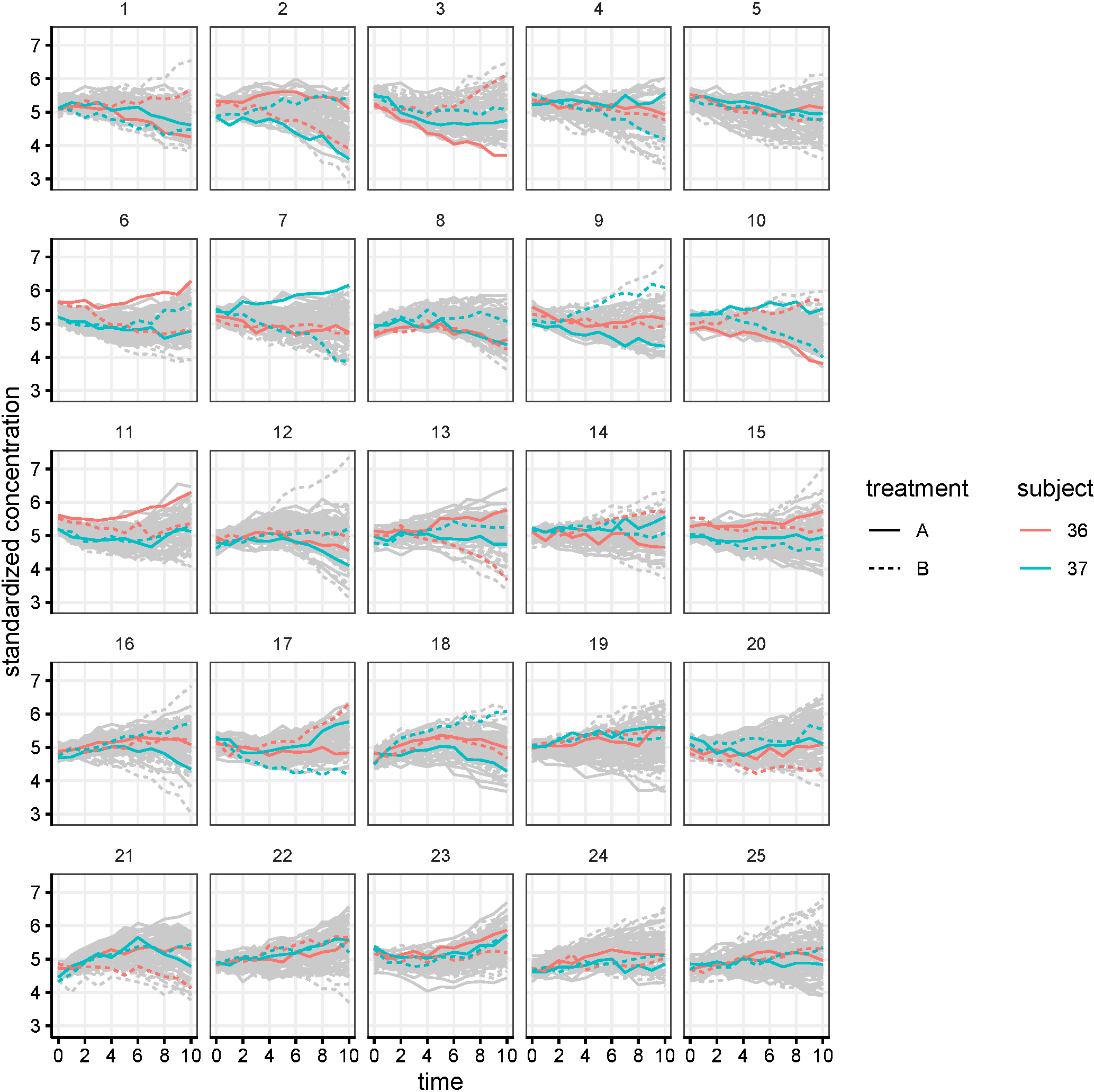

Supplement: S4 Fig — (TIF) [file pcbi.1011221.s004.tif]

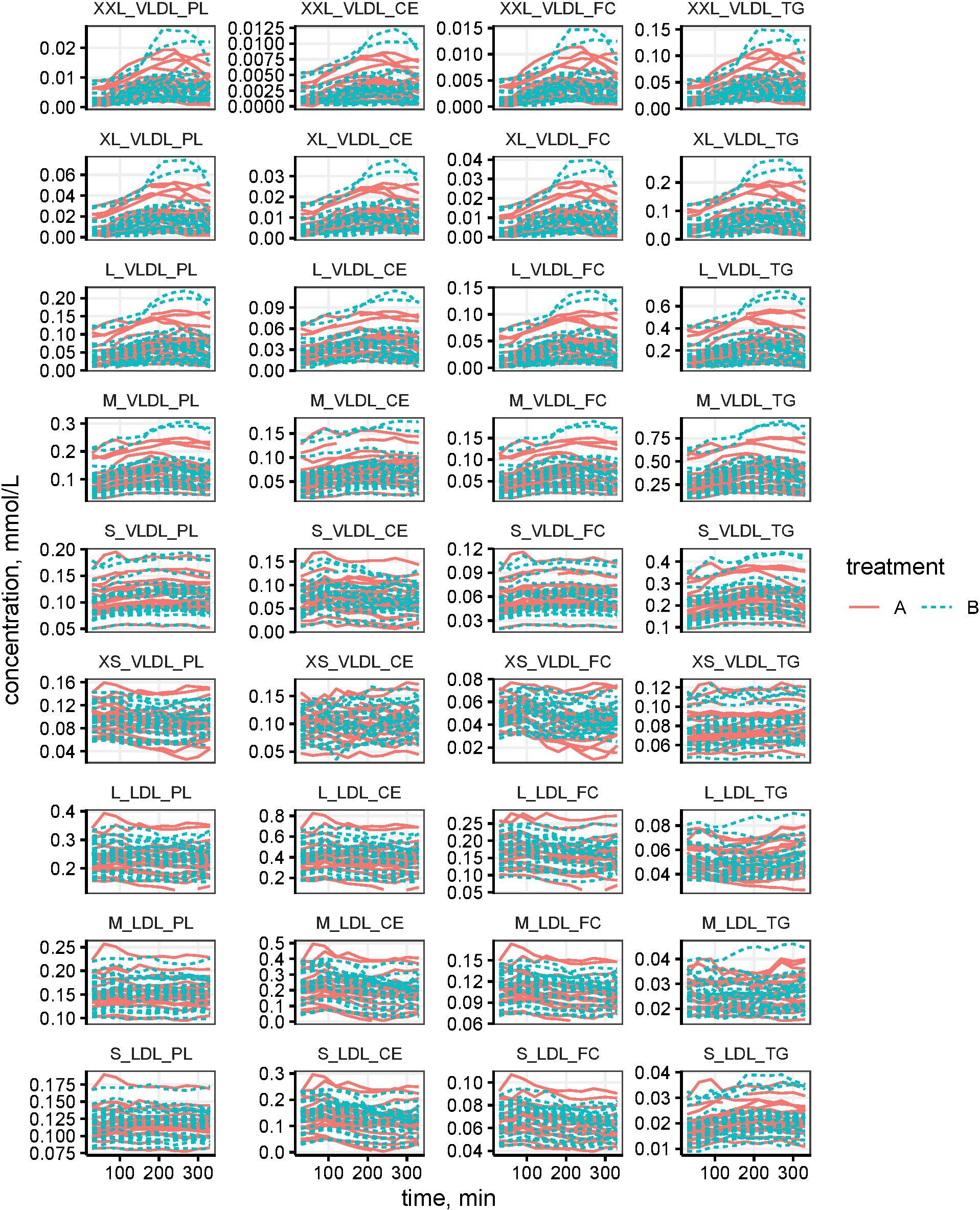

Supplement: S5 Fig — XXL: extra extra large, XL: extra large, L: large, M: medium, S: small, XS: extra small. (TIF) [file pcbi.1011221.s005.tif]

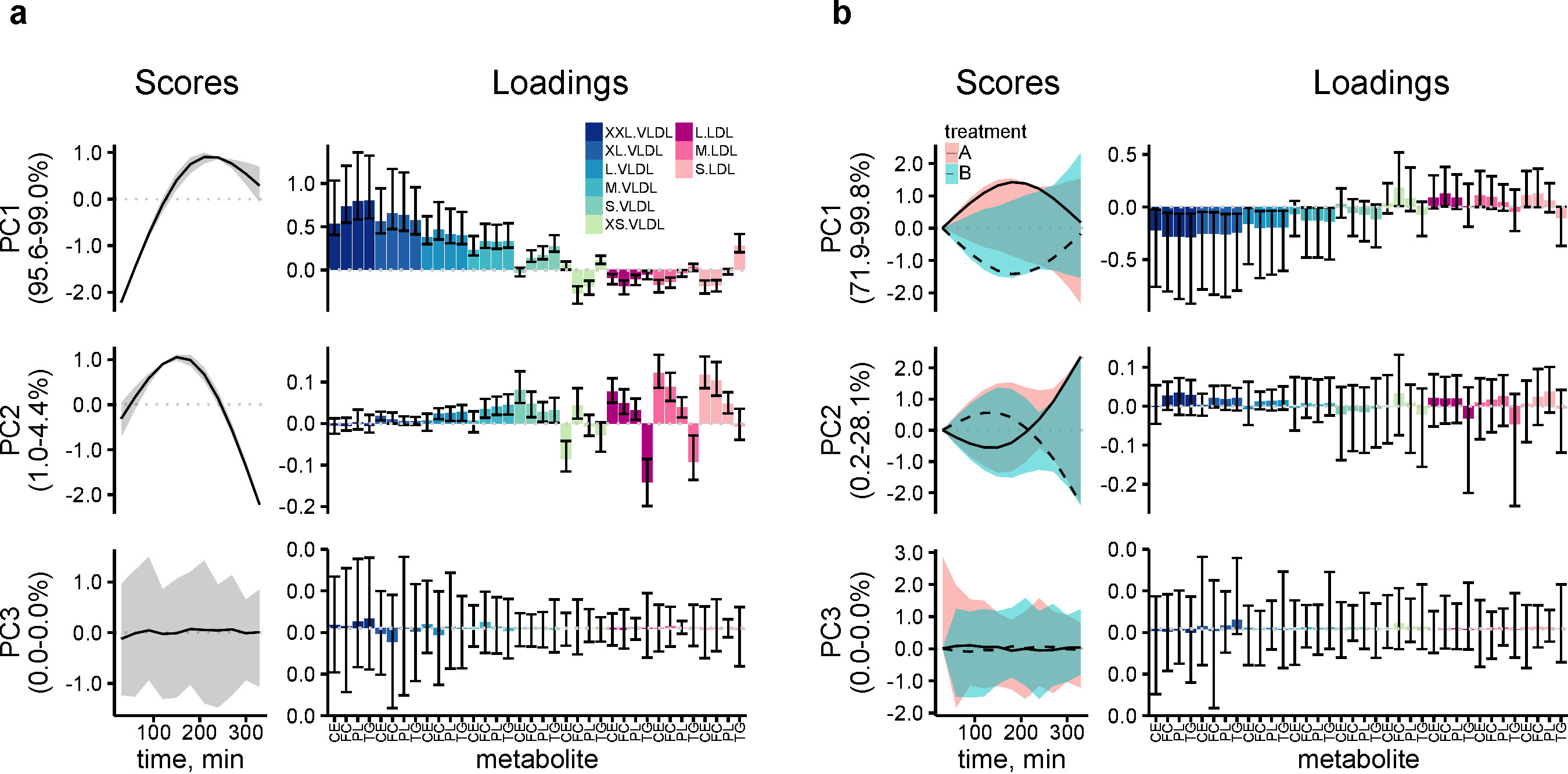

Supplement: S6 Fig — Scores contain the prominent patterns over time, while the loadings show the association of the scores with the metabolite time-courses. Metabolites are shown in the axis label of the loadings with the colours indicating the various subclasses. FC: free cholesterol, CE: esterified cholesterol, PL: phospholipids, TG: tryglicerides. Resampling based 95% confidence intervals are shown as shaded area for the scores and error bars for the loadings. (TIF) [file pcbi.1011221.s006.tif]

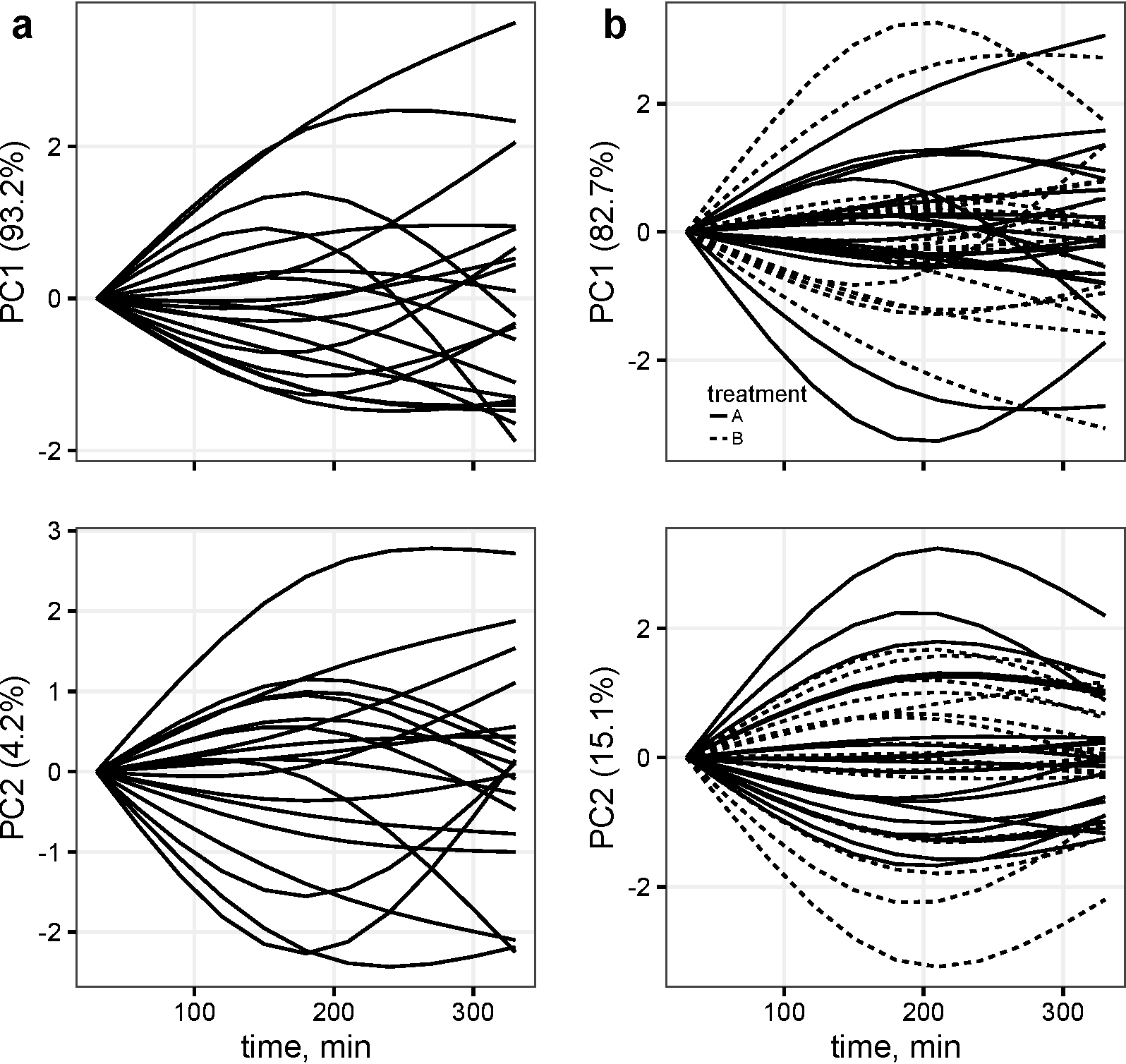

Supplement: S7 Fig — (TIF) [file pcbi.1011221.s007.tif]

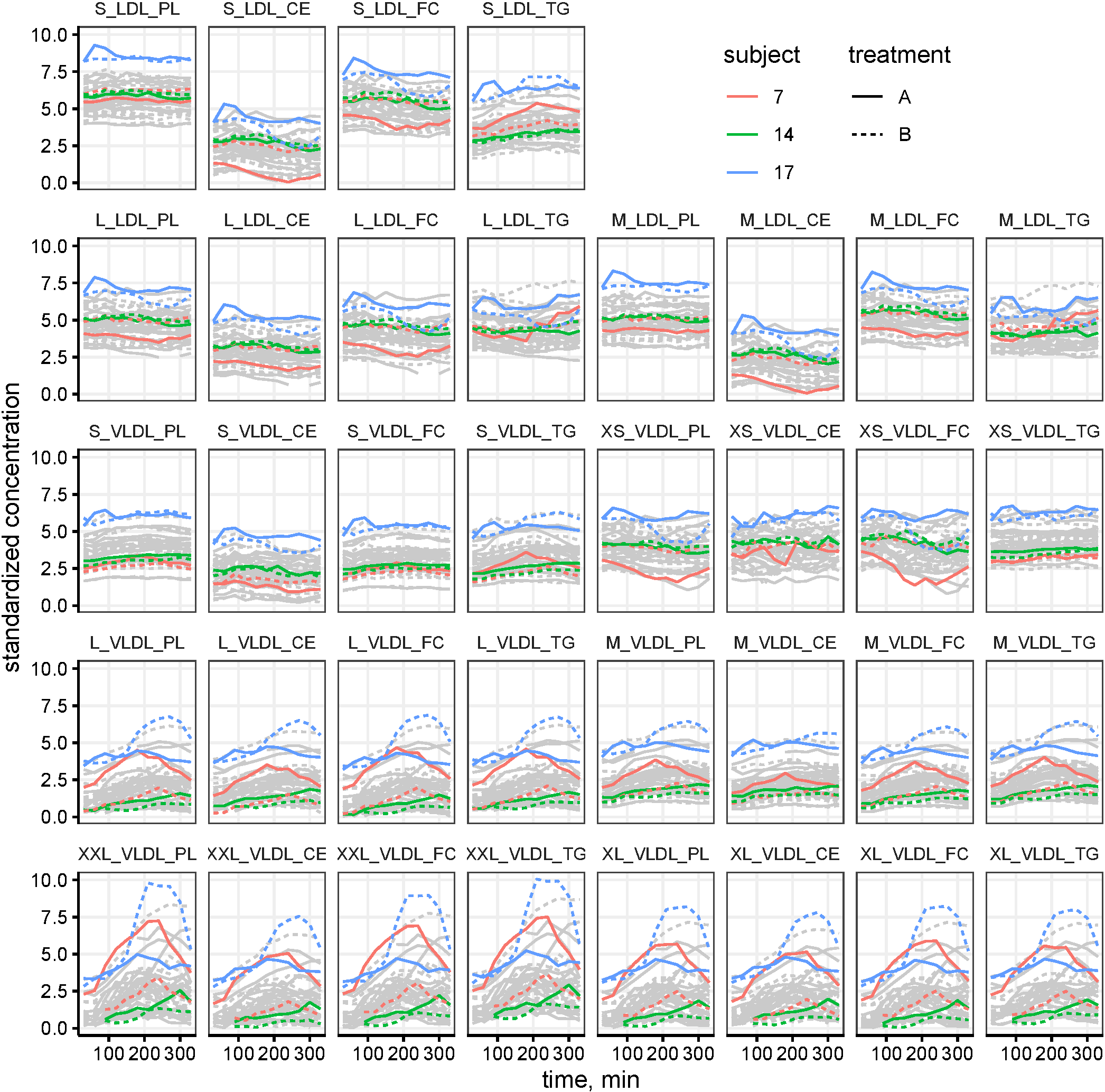

Supplement: S8 Fig — XXL: extra extra large, XL: extra large, L: large, M: medium, S: small, XS: extra small. (TIF) [file pcbi.1011221.s008.tif]
